# Mitochondrial P-JNK target, SAB (SH3BP5), in regulation of cell death

**DOI:** 10.3389/fcell.2024.1359152

**Published:** 2024-03-15

**Authors:** Sanda Win, Tin Aung Than, Neil Kaplowitz

**Affiliations:** Department of Medicine, Division of Gastroenterology and Liver Diseases, University of Southern California, Los Angeles, CA, United States

**Keywords:** apoptosis, ferroptosis, necrosis, pyroptosis, SAB (SH3BP5), Jun N-terminal kinase, JNK-SAB-ROS activation loop, SAB-KIM1 peptide

## Abstract

Cell death occurs in various circumstances, such as homeostasis, stress response, and defense, *via* specific pathways and mechanisms that are regulated by specific activator-induced signal transductions. Among them, Jun N-terminal kinases (JNKs) participate in various aspects, and the recent discovery of JNKs and mitochondrial protein SAB interaction in signal regulation of cell death completes our understanding of the mechanism of sustained activation of JNK (P-JNK), which leads to triggering of the machinery of cell death. This understanding will lead the investigators to discover the modulators facilitating or preventing cell death for therapeutic application in acute or chronic diseases and cancer. We discuss here the mechanism and modulators of the JNK-SAB-ROS activation loop, which is the core component of mitochondria-dependent cell death, specifically apoptosis and mitochondrial permeability transition (MPT)-driven necrosis, and which may also contribute to cell death mechanisms of ferroptosis and pyroptosis. The discussion here is based on the results and evidence discovered from liver disease models, but the JNK-SAB-ROS activation loop to sustain JNK activation is universally applicable to various disease models where mitochondria and reactive oxygen species contribute to the mechanism of disease.

## Introduction

Mitochondrial-dependent regulated cell death was first described in apoptosis and mitochondrial permeability transition (MPT)-driven necrosis. Jun N-terminal kinases (JNKs) contribute as a key stress kinase to modulate intrinsic and extrinsic pathways of apoptotic cell death. Mammalian JNKs are expressed by three distinct genes, *JNK1-3*. Multiple splice variants form a family of kinases phosphorylating serine adjacent to the proline of target proteins in response to various forms of cellular and metabolic stress, growth factors, and inflammatory cytokines. JNK-interacting protein (JIP) provides a cytoplasmic platform for JNK docking at kinase-interacting motif (KIM) to interact and phosphorylate JNK-targeted proteins (Liu and Lin, 2005; Dhanasekaran and Reddy, 2008). JNK binding to mitochondrial scaffold protein SAB (SH3BP5) was first described in chick embryonic fibroblasts by co-localization of activated P-JNK and SAB in immuno-fluorescence staining (Wiltshire et al., 2008). Importantly, SAB is a key mediator in JNK regulation of mitochondrial-dependent cell death and was first described in numerous *in vitro* and *in vivo* models such as galactosamine (GalN)/tumor necrosis factor (TNF)α-induced apoptosis, lipotoxicity or endoplasmic reticulum (ER) stress-induced apoptosis, and acetaminophen-induced necrosis (Win et al., 2011; Win et al., 2014; Win et al., 2015). There are several additional modes of cell death which are categorized by the Nomenclature Committee on Cell Death (NCCD) in 2018 (Galluzzi et al., 2018). This review, however, focuses on modes of cell death which are specifically regulated by the interaction of JNK and outer mitochondrial membrane (OMM) protein SAB.

## The pivotal role of SAB in JNK translocation to mitochondria

**FIGURE 1:**
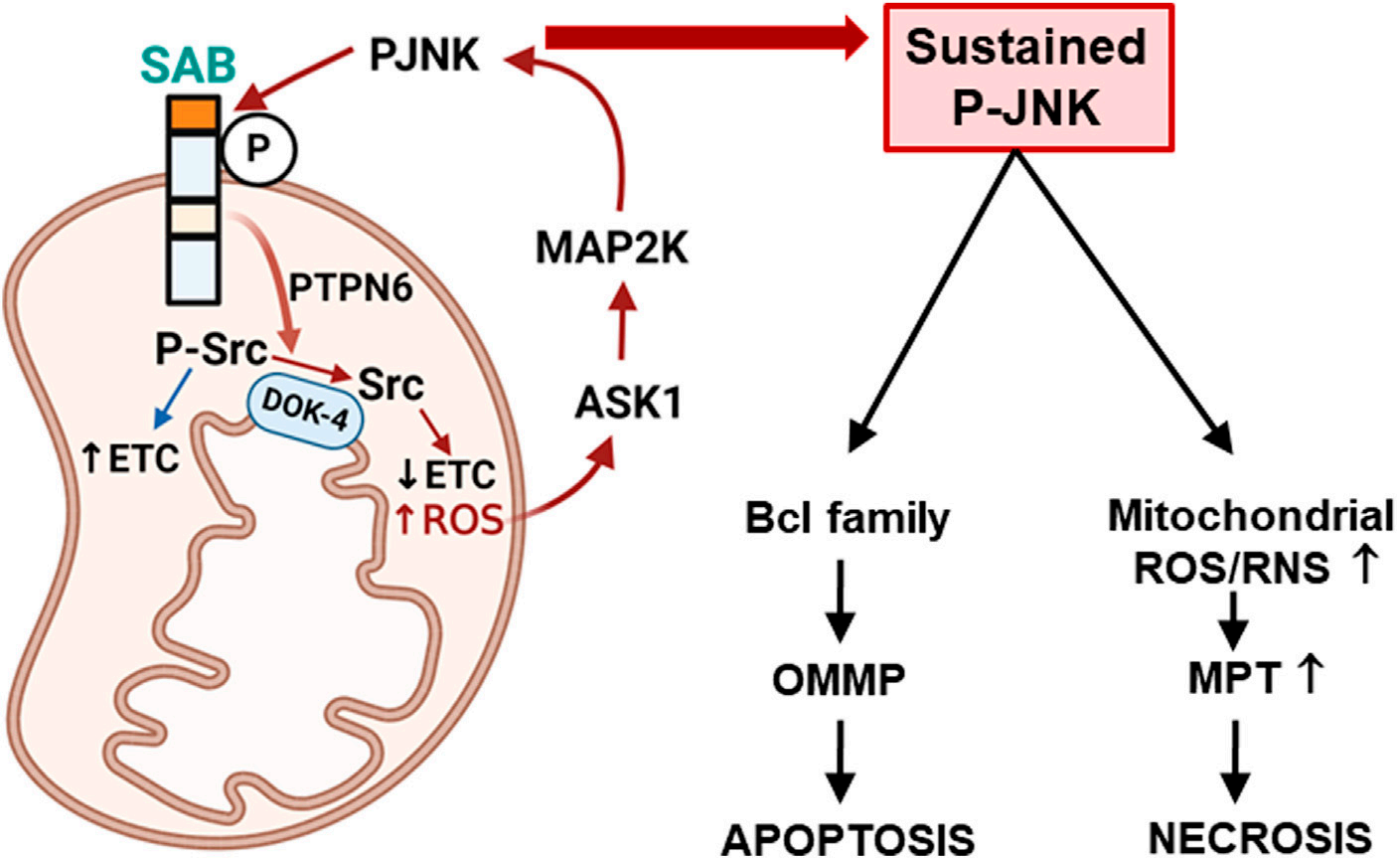
Topology of SAB and SAB regulation of apoptosis or necrosis *via* mitochondrial ROS production and sustained P-JNK. Topology of SAB is depicted in the figure. SAB is located at the outer mitochondrial membrane (OMM) with one membrane-spanning sequence. C-terminus of SAB is facing the cytoplasm and has JNK-docking motif (KIM2, dark orange) and JNK phosphorylation sites. N-terminus of SAB is facing the intermembrane space and has the SH3 domain-binding sequence (light orange) which may harbor PTPN6. Stress-induced activated JNK (P-JNK) binding and phosphorylation of OMM protein SAB lead to the release of intramitochondrial protein tyrosine phosphatase PTPN6, which then translocates to inner mitochondrial protein DOK4 where active P-Src is dephosphorylated and inactivated by PTPN6. Activated P-Src maintains the mitochondrial electron transport chain (ETC) activity, and dephosphorylation of Src decreases ETC and increases ROS production, which upregulates ASK1 activation and then MAP2K and JNK activation. The JNK-SAB-ROS feed forward activation loop sustains the JNK activation and incrementally increases ROS production, leading to apoptosis via outer mitochondrial membrane permeabilization (OMMP) or necrosis via increased mitochondrial permeability transition (MPT) depending on the nature of the specific cell death inducer.

SAB, abbreviated from SH3 domain-binding protein that preferentially associates with Bruton’s tyrosine kinase (BTK), was first identified in 1998 as a binding protein of the SH3 domain of BTK using Far-Western blotting. SAB was then cloned from a cDNA library made from the human placenta (Matsushita et al., 1998). Later, SAB was identified in yeast two-hybrid screening of human HeLa cell cDNA library using rat JNK3 as bait, and the SAB gene was officially named as SH3 domain-binding protein 5 (SH3BP5) in 2002 (Wiltshire et al., 2008). JNK, specifically activated JNK (P-JNK), binding of SAB was confirmed by GST pull-down assay of truncated expressed SAB protein. Sub-cellular localization of SAB on mitochondria was described in chick embryonic fibroblast by immuno-fluorescence staining of SAB, and it was found to co-localize with anisomycin-activated P-JNK on mitochondria (Wiltshire et al., 2008). One SAB gene expresses two splice variants: longer transcript isoform (a) and shorter transcript isoform (b). Two independent investigators simultaneously uncovered the functional role of SAB in JNK-mediated regulation of cell death in 2011 (Chambers and LoGrasso, 2011; Win et al., 2011). Subsequently, many investigators have found JNK translocation to mitochondria when JNK is activated, and mounting evidence has indicated that SAB is the only JNK docking protein on mitochondria. Adenoviral shRNA-mediated depletion of SAB in the liver completely inhibits P-JNK translocation to mitochondria (Win et al., 2011). The level of mitochondrial residence protein SAB directly correlates to the level of translocated P-JNK on mitochondria (Win et al., 2019). Intriguingly, p38 and P-p38 localization and level of mitochondria are independent of SAB expression (Zhang et al., 2017). Moreover, SAB does not directly associate and interact with MAP2Ks (MKK4 and 7) and MAP3Ks (Zhang et al., 2017). Differential expression of SAB in adult male and female mice and humans was reported, and mitochondria from female mice have decreased P-JNK translocation because of low levels of SAB protein. Increased expression of SAB causes more P-JNK translocation (Win et al., 2019). In addition to previous studies, subcellular fractionation of the liver confirmed that SAB is almost exclusively in mitochondria fraction but not in ER, cytosol, and nuclear fraction. Notably, the subcellular distribution of SAB does not change during toxic stress conditions (Win et al., 2011). Importantly, the overexpression of human and mouse SAB confirmed the mitochondrial localization of SAB (Win et al., 2019). SAB resides in the OMM as confirmed by the proteinase K digestion assay. Using N-terminal- and C-terminal-specific SAB antibodies, the N-terminus of SAB is found exposed to the intermembrane space, whereas the C-terminus of SAB is exposed to the cytoplasm (Figure 1). One membrane-spanning domain was predicted (Win et al., 2016).

The N-terminus of SAB is speculated to have four coiled-coil domains, and SAB is a homolog of Ras-related GTP-binding protein Rab11 (RAB-11)-interacting protein-1 (REI-1) and a guanine nucleotide exchange factor (GEF) of *Citrobacter elegans*; however, both SAB and REI-1 do not contain any known Rab-GEF domain. REI-1 is expressed in *C. elegans* germline and co-localizes with small guanosine triphosphate hydrolase (GTPases) RAB-11 on the late-Golgi membranes (Sakaguchi et al., 2015; Sakaguchi et al., 2016; Sato et al., 2016). Given this, the structural analysis revealed the Rab-11 binding site on both helix α1 and α4 of murine SH3BP5 and REI-1 of *C. elegans* (Jenkins et al., 2018). Recently, co-localization of SAB, RAB-11, and lysosome marker Lamp1 in HeLa cells was described, but SAB and RAB-11 co-localization is much greater than Lamp1 distribution (Goto-Ito et al., 2019) because SAB is overwhelmingly found in the OMM. Overall, these results implied that the regulation of RAB-11 by SAB in the context of JNK-mediated cell death is an intriguing view that deserves further investigation. The C-terminus of SAB exposed to cytoplasm has a JNK docking site, referred to as KIM2, and serine phosphorylation sites. The other predicted docking site KIM1 is submerged in the mitochondrial membrane, and the coiled-coil region of SAB is predicted to lie in the intermembrane space (Win et al., 2016). With these various aspects, there is plenty of area of research to explicitly illuminate how SAB participates in small GTPase regulation of mitochondrial membrane transport and morphology in JNK-mediated cell deaths.

## Crucial role of SAB in sustained activation of JNK

JNK is activated by numerous conditions of organelles, cellular and metabolic stress/injury, and growth and proliferation conditions. Importantly, JNK-mediated cell death is directly correlated with the sustained activation mechanism of JNK (Dhanasekaran and Reddy, 2008). Through the mitogen-activated protein kinase (MAPK) cascade, JNK, which is one of two MAPKs, is phospho-activated by MAP2K, MKK4, and MKK7, which are regulated by MAP3K such as apoptosis signal-regulating kinase 1 (ASK1), mixed lineage kinases (MLKs), and transforming growth factor-β (TGF-β)-activated kinase 1 (TAK1). Subcellular localization and structurally associated proteins determine the activation of MAP3K in various cellular conditions. Numerous pieces of evidence suggest that ASK1 and MLKs predominantly contribute to the sustained activation of JNK. The structure of ASK1 defines the role in the redox activation of JNK. ASK1 has a kinase domain at the center and one coiled-coil domain on either side of the kinase domain. The N-terminal domain is associated with thioredoxin and TNF receptor-associated factors (TRAFs) (Weijman et al., 2017). The C-terminus of ASK1 has a 14-3-3 binding site (Cockrell et al., 2010), followed by a constitutive oligomerization region. ASK1 is silent when homo-oligomerization occurs through the C-terminal coiled-coil region in non-stress conditions (Kawarazaki et al., 2014). Dissociation of thioredoxin from ASK1 under oxidative stress leads to the association of ASK1 and TRAF, leading to oligomerization of N-terminus and activation of ASK1 through autophosphorylation of Thr845 located in the kinase domain (Cebula et al., 2015). Since mitochondrial thioredoxin-2 has been identified, mitochondrial localization of ASK1 was proposed, but further investigations are required (Zhang et al., 2004). ASK1 protein itself is degraded in oxidative stress *via* ubiquitination, and inhibition of ubiquitination or facilitating de-ubiquitination stabilizes the ASK1 activity (Nagai et al., 2009). In addition, ASK1 activity is well regulated by cellular FLICE-like inhibitory protein (cFLIP), which competitively inhibits TRAF binding to ASK1 and prevents ASK1 dimerization and activation (Wang et al., 2017a). However, degradation of cFLIP is mediated by ubiquitination by itchy E3 ubiquitin protein ligase (ITCH,) which is activated by JNK (Chang et al., 2006). Recent evidence suggests that caspase recruitment domain protein 6 (CARD6) suppresses ASK1 phospho-activation but not TAK1, indicating the selectivity of regulation (Qin et al., 2018). TAK1 is phosphorylated and activated *via* the TLR/IL1 receptor and TRAFs, leading to the activation of NF-κB and JNK *via* IKK and MAP2K, respectively (Takaesu et al., 2001). Hepatocyte-specific deletion of TAK1 is lethal, suggesting the hepatoprotective role of TAK1 (Inokuchi et al., 2010), although further exploration is required. MLKs are regulated by small GTPases, CDC42 and RAC1 (Kelkar et al., 2005). Signal integration of SRC tyrosine kinase, RAC GTPase, and MLKs on the JIP1 platform activates MKK7 and JNK in mouse embryonic fibroblast (MEF) cells (Morel et al., 2010). JIP is known as a JNK-specific scaffold protein, and the pathway selectively activates JNK but not p38.

The increased understanding of the regulation of MAP3K activation enables us to construct the mechanism of sustained activation of JNK depending on the context. *In vivo* GalN/TNFα or *in vitro* ActD/TNFα induced apoptosis; initial receptor-mediated activation of ASK1 and JNK is sustained when the antioxidant system is blocked by GalN or ActD. Furthermore, deletion of SAB completely prevents sustained activation of JNK and cell death, suggesting the crucial role of SAB to sustain JNK activation (Win et al., 2011). Of note, depletion of mitochondrial SAB does not interfere with the initial activation of JNK such as receptor-mediated activation by ASK1 or JIP-mediated activation by MLKs. A compelling body of evidence indicates that the JNK-ITCH-ASK1 signal activation axis does not have an effect in contexts where SAB is deleted because depletion of SAB or MKK4/7 completely prevents TNFα-induced JNK activation and cell death, indicating the requirement of MAP2K, JNK, and SAB to sustain ASK1 and JNK activation (Figure 1) (Win et al., 2011; Win et al., 2016; Zhang et al., 2017). Furthermore, SAB contributes a primary role in the sustained activation of JNK in lipotoxicity, ER stress-induced apoptosis, and acetaminophen-induced necrosis, which we discuss below (Win et al., 2011; Win et al., 2014; Win et al., 2015; Win et al., 2016; Win et al., 2019).

## Essential role of SAB and JNK interaction in mitochondrial superoxide production

Mitochondria produce superoxide generated by electrons leaking from the electron transfer system located in the inner membrane of mitochondria. Production of mitochondrial superoxide in a cell is much more abundant than that in other systems such as xanthine oxidase and nicotinamide adenine dinucleotide phosphate (NADPH) oxidase. Superoxide generated from mitochondria is a major cause of cellular oxidative damage (Brand et al., 2004). Until recently discovered by Win et al. (2016), there has been a knowledge gap in understanding how stress-induced JNK activation upregulates superoxide production from mitochondria. Stress-induced JNK activation causes translocation and binding of P-JNK to OMM protein SAB. P-JNK binds to the JNK docking site (KIM2) located at the C-terminus of SAB which is exposed to the cytoplasm. Mutation of the KIM2 sequence completely prevents P-JNK translocation and binding to mitochondria. SAB is also a substrate of JNK and presumably phosphorylated at serine 421 (human) or serine 424 (mouse) in the C-terminus facing the cytoplasm; mutation of this serine completely prevents the JNK activation-mediated liver injury in GalN/TNFα and acetaminophen hepatotoxicity. Therefore, SAB and P-JNK interaction on the OMM is the essential initial step for the production of superoxide from mitochondria. Activated P-JNK only binds to SAB, and P-JNK does not enter the mitochondria. Under basal conditions, the N-terminus of SAB including the SH3-domain-binding site which is exposed to the intermembrane space is associated with protein tyrosine phosphatase non-receptor type 6 (PTPN6/SHP1). SAB then releases PTPN6 when P-JNK binds and phosphorylates the C-terminus of SAB. Tyrosine-protein kinase c-SRC, mainly in the active form P-419-SRC, is inside mitochondria and is required to maintain the function of the electron transport chain (Salvi et al., 2002; Miyazaki et al., 2003; Tibaldi et al., 2008; Foster et al., 2009; Ogura et al., 2012). PTPN6 release from SAB leads to dephosphorylation of activated P-419-Src, which occurs on and requires the platform, docking protein 4 (DOK4), located on the mitochondrial inner membrane (Figure 1) (Win et al., 2016). Decreased P-Src inhibits mitochondrial respiration and enhances reactive oxygen species (ROS) production (Win et al., 2014). One can speculate that when the translocation of P-JNK to mitochondria is little, such as in postprandial hepatocytes, the recovery of mitochondrial respiration is faster and thus ROS production reduces. Conclusively, the KIM1 peptide completely blocks the interaction of SAB and JNK, and prevents superoxide production from mitochondria and cell death (Chambers et al., 2011a; Chambers and LoGrasso, 2011; Win et al., 2014). SAB functions similarly to a membrane receptor which transduces the P-JNK effect on the surface of mitochondria to signal transduction inside the mitochondria.

## SAB is a key mediator in mitochondrial-dependent cell death

### SAB regulation of apoptosis and necrosis

To explicitly illuminate the critical contribution of JNK and SAB interaction, and how interaction leads to sustaining JNK activation and regulation of apoptosis and necrosis, Win et al. examined the chronological and spatial changes of JNK in GalN/TNFα or ActD/TNFα-induced extrinsic-apoptosis model, lipotoxicity or ER stress-induced intrinsic-apoptosis model, and acetaminophen-induced MPT-driven necrosis model (Win et al., 2011; Win et al., 2014; Win et al., 2015; Win et al., 2016; Zhang et al., 2017; Win et al., 2019). In these various models of apoptotic and necrotic modes of cell death, it all starts with initial JNK activation, and the primary role of SAB is to increase mitochondrial superoxide production, resulting in sustained activation of JNK (P-JNK) leading to the activation of stress-inducer-specific cell death machinery such as modulating activity or expression of Bcl2 family proteins in TNFα-induced apoptosis, transcriptional upregulation of p53-upregulated modulator of apoptosis (PUMA) in lipotoxicity, Ca^2+^ influx to mitochondria in tunicamycin-induced cell death, massive depletion of glutathione (GSH), and the burst of ROS in a narrow time frame followed by the collapse of mitochondrial bioenergetics due to mitochondrial permeability transition pore (MPTP) in acetaminophen-induced necrosis. Therefore, the effect of unique biochemical properties of stress-inducers must be considered in modeling the regulation of the cell death mechanism.

Initial JNK activation: JNK is a stress-responsive kinase and is activated in all bio-physical-chemical changes of cell and cellular environment *in vitro* or *in vivo*; however, JNK kinase activity including P-JNK may be undetectable in physiological circumstances because of its transient nature and weak activity. Initial JNK activation occurs when a stress-inducer first interacts with the cell: TNFα engagement to the death receptor activates JNK in 5–10 min and diminishes rapidly in 30 min (Win et al., 2011), exposure of free fatty acid to the cell membrane activates membrane-associated c-Src and then JNK is activated transiently and detected with the kinase assay (Holzer et al., 2011), the non-lethal dose of tunicamycin-induced ER stress activates JNK in 5–10 min and diminishes quickly in 30 min (Brown et al., 2016), the first kinase of MAPK cascade activated by acetaminophen is MLKs, and non-lethal dose (150 mg/kg i.p) of acetaminophen activates cytosol JNK in 15–30 min and diminishes in an hour (Sharma et al., 2012). Thus, initially activated P-JNK becomes sustained and higher when P-JNK translocates and interacts with mitochondrial SAB.

Sustained JNK activation *via* the JNK-SAB-ROS activation loop: A mitochondrion is a complete hub where ROS is produced, and where MAPK cascade gathers to amplify and sustain JNK activation. We discuss here the principle of the mechanism (Figure 1) of sustained activation of JNK using acetaminophen and GalN/TNFα model because of the detailed examination of numerous independent investigators in the field. Initial activated P-JNK translocates to mitochondria where P-JNK binds and phosphorylates SAB. P-JNK co-immunoprecipitated with SAB shortly after toxic stress before the liver injury occurred (Win et al., 2011; Liu et al., 2015; Heslop et al., 2020). This is further supported by the finding that knockdown of *SAB* prevented JNK translocation to mitochondria, inhibited sustained activation of JNK, and protected cell death *in vitro* and *in vivo* models (Win et al., 2011; Win et al., 2014; Win et al., 2015). Furthermore, hepatocyte-specific deletion of *SAB via* the delivery of AAV8-TBG-Cre to *SAB*-floxed mice or delivery of tamoxifen to *SAB*-floxed mice crossed with transgenic tamoxifen-inducible Alb-Cre mice completely protects against hepatic apoptosis or necrosis in those models. The depletion of SAB completely prevents translocation of JNK to mitochondria, suggesting that SAB is the only identifiable JNK docking mitochondrial-resident protein (Win et al., 2016). The depletion of SAB does not inhibit the association of mitochondria and p38 (Zhang et al., 2017); however, there is a report showing the phosphorylation of SAB by JNK and p38 in a cell-free system (Court et al., 2004). The topology and structure of SAB were revealed recently by several investigators (Win et al., 2016; Jenkins et al., 2018). The N-terminus of SAB facing intermembrane space has four coiled-coil domains and an SH3-domain-binding domain. The C-terminus of SAB faces the cytoplasm and contains two kinase-interacting motifs (KIMs) where JNK docks to its substrates. One membrane-spanning sequence is predicted, and only the shorter C-terminus of SAB exposed to the cytoplasm is removed in the proteinase K digestion assay, implying that longer N-terminus of SAB is intramitochondrial. Exposure of isolated mitochondria with P-JNK dose-dependently inhibits mitochondria respiration and decreases activated Src (P-419-Src) in mitochondria, but these did not occur in mitochondria from the SAB knockout liver. P-JNK inhibition of respiration was prevented by the KIM-blocking peptide (Win et al., 2014; Win et al., 2015; Win et al., 2016). P-419-Src is required to maintain the activity of the electron transport chain (Salvi et al., 2002; Miyazaki et al., 2003; Tibaldi et al., 2008; Foster et al., 2009; Ogura et al., 2012). Inactivation of mitochondria Src occurred in both apoptosis (TNF/GalN) and necrosis (APAP) models. Further investigation revealed that P-JNK binding and phosphorylation to SAB cause a release of protein tyrosine phosphatase non-receptor type 6 (PTPN6/SHP1), which is associated at the N-terminus of SAB, leading to dephosphorylation of activated Src on the DOK4 platform. DOK4 is found exclusively in the mitochondria fraction and is associated with the inner mitochondrial membrane (IMM). Knockdown of PTPN6 or DOK4 does not affect SAB levels but phenocopies the effects of SAB knockdown or knockout in hepatocyte response to stress (Win et al., 2016).

The next crucial step is the effect of the interaction of P-JNK with SAB on mitochondrial ROS production. Using isolated normal liver mitochondria, exposure of recombinant P-JNK1 and/or 2 leads to the inhibition of oxidative phosphorylation and maximum respiratory capacity, and production of superoxide in the presence but not in the absence of ATP, suggesting that phosphorylation of SAB was required. Because ROS production was inhibited in liver mitochondria isolated from SAB knockout mice or normal mitochondria treated with KIM1 peptide which blocks JNK and SAB interaction, SAB is required for mitochondrial ROS production (Win et al., 2014; Win et al., 2015; Win et al., 2016; Huo et al., 2017). ROS production is facilitated in ER stress due to Ca^2+^ influx which upregulates mitochondrial metabolism and NADH production (Win et al., 2014).

Released ROS oxidizes thioredoxin-relieving inhibition of ASK1 dimerization. Facilitating ASK1 N-terminal dimerization allows self-activation of ASK1 (Zhang et al., 2018). Activated ASK1 activates MKK4/7, which then activates JNKs (Zeke et al., 2016; Win et al., 2018a; Win et al., 2018b). MLKs are also activated by ROS *via* the activation of plasma membrane-associated Src. Depletion of ASK1 or MLK2/3 completely prevents sustained activation of JNK and APAP-induced liver necrosis. Furthermore, P-MKK4 is associated with mitochondrial P-JNK (Zhang et al., 2017). Depletion or inhibition of MLK2/3 prevents initial JNK activation in APAP toxicity but deletion or inhibition of ASK1, MKK4, or SAB does not, suggesting that MLK2/3 is the first MAP3K activated, and ASK1, MKK4, and SAB are involved in sustained activation of JNK (Urano et al., 2000; Liang et al., 2006; Holzer et al., 2011; Win et al., 2011; Papa et al., 2019). Therefore, the feed-forward activation of sustained JNK activation occurs through the ASK1-MKK4-JNK-SAB-ROS activation loop (Figure 1), which is a key player in the mechanism of cellular stress and damage (Win et al., 2016; Win et al., 2018a).

The duration and degree of sustained JNK activation mediate many consequences, both through upregulation of activating protein-1 (AP-1) targets (Win et al., 2018a) involved in the proliferation; inflammation (production of cytokines and chemokines); metabolic gene dysregulation, for example, gene repressors, such as NCOR1 suppression on peroxisome proliferator-activated receptor alpha (PPARα) and thioredoxin-disulfide reductase (TR); or enhanced apoptosis through the direct activation of proapoptotic protein BAX, and inhibition of anti-apoptotic Bcl2 family members such as Bcl-X_L_, Bcl2, or Mcl-1.

### Potential role of SAB in the regulation of ferroptosis and pyroptosis

Ferroptosis: Ferroptosis is one of the mechanisms of stress-driven regulated cell death (RCD) of cancer cells and non-cancer cells, leading to severe lipid peroxidation as a consequence of Fenton reaction (iron and H_2_O_2_ reaction generating hydroxyl or hydroperoxyl radicals) when cellular GSH synthesis and GSH is impaired (Galluzzi et al., 2018). Cells committed to ferroptosis exhibit necrotic morphology and mitochondrial alterations encompassing shrinkage, reduced cristae, and ruptured OMM. The ferroptosis mechanism is independent of caspases and necrosome formation, and cannot be prevented by cyclophilin D inhibitor, suggesting the distinct RCD mechanism from apoptosis, necroptosis, and necrosis. Ferroptosis is enhanced by inducers such as erastin, RSL3, and FIN56 and is prevented by iron chelators, ferrostatins, and lipid peroxidation inhibitors such as liproxstatins. Intracellular (cytoplasmic and mitochondrial) reduced glutathione (GSH) is critically important in preventing ferroptosis because GSH is a co-factor of glutathione peroxides (GPx isoforms such as GPx1 and 4) for the removal of radicals generated from H_2_O_2_ and lipid peroxides. Thus, depletion or inhibition of GPx commits cells to ferroptosis inducer-induced cell death. Induction of ferroptosis enhances anticancer drug susceptibility and thus becomes an avenue for anticancer treatment. The mechanism of ferroptosis in biology and pathological processes and the anticancer therapeutic application of ferroptosis inducers have been reviewed thoroughly (Xie et al., 2016; Wu et al., 2021; Yan et al., 2021).

Lipid (mainly PUFA) peroxidation is a key step in committing to ferroptosis. The subcellular location of the initial event of lipid peroxidation may be from mitochondria, ER, or plasma membrane, and further experiments are required to clarify signal transduction pathways, leading to ROS production and lipid peroxidation. There are multiple sources of ROS in the cell including nicotinamide adenine dinucleotide phosphate (NADPH) oxidase (NOX), xanthine oxidase (XO), uncoupling of nitric oxide synthase (NOS), cytochrome P450, and inhibition of mitochondrial electron transport chain (ETC), and more than one source of ROS could be responsible for lipid peroxidation in ferroptosis. For instance, the RAS-mutant tumor cell, Calu-1, which expresses high NOX1, is protected from erastin-induced ferroptosis by NOX inhibitors, but human fibrosarcoma-derived epithelial cells, HT-1080 cells, are only partially protected by NOX inhibitors. In this context, the early role of mitochondrial ROS production needs further clarification. Mitochondrial O_2_^−^ production and NOX activation cross-talk have been shown in several models (Dikalov, 2011); serum withdrawal of HEK293T cells activates mitochondrial O_2_^−^ production within a few minutes and triggers NOX1 activation 4–8 h later (Lee et al., 2006). Angiotensin II-induced NOX2 activation and O_2_^−^ production in endothelial cells *via* membrane-bound c-Src are reversed by mitochondrial SOD2 overexpression or mitochondrial-targeted antioxidant (Mito-TEMPO) treatment but enhanced by depletion of SOD2 (Dikalova et al., 2010). Based on the published evidence, a concrete approach is required to address the notion that the activation of NADPH oxidases increases the production of mitochondrial ROS and *vice versa* in ferroptosis of cell culture and disease models such as ischemia-reperfusion injury and HCC.

Notably, the expression of NOX genes (1–5) in nontumor cells is cell/tissue-specific. In the liver, hepatocytes, hepatic stellate cells, and endothelial cells express NOX1, 2, and 4, respectively. Kupffer cells express NOX2, whereas inflammatory cells such as monocytes/macrophages express NOX1 and 2. It is important that NOX enzymes have different subcellular localization but also generate distinct ROS; NOX1 and 2 generate O_2_^−^, NOX4 produces basal H_2_O_2_, and NOX5 produces H_2_O_2_ in a Ca^2+^-dependent mechanism (Dikalov et al., 2008; Guzik et al., 2008; Takac et al., 2011). However, mitochondrial localization of NOX is unlikely and thoroughly discussed in another review (Dikalov, 2011). A recent study implied that mitochondria contribute to doxorubicin (DOX)-induced ferroptosis and cardiotoxicity (Tadokoro et al., 2020). DOX compared to erastin and RSL3 is a unique ferroptosis inducer, causing lipid peroxidation in mitochondria but not in other organelles because of the formation of the DOX-Fe^2+^ complex in mitochondria. DOX is known to activate apoptotic caspase activation in a quicker time (peak at 10 h). GPx4 was gradually downregulated, and lipid peroxidation increased until 30 h. Caspase inhibitor and Fer-1 partially prevent DOX-induced cell death, whereas a combination (Tadokoro et al., 2020) or Mito-TEMPO (Fang et al., 2019) fully prevents cell death. Another mitochondrial-targeted antioxidant, nitroxide XJB-5-131, also inhibits erastin or RSL3-induced ferroptosis (Krainz et al., 2016). Overall, these and other results implied that the regulation of mitochondrial ROS production contributes to the activation of the ferroptosis mechanism (Guo et al., 2022; Oh et al., 2022; Liu et al., 2023). Therefore, further investigation into ferroptosis is needed to delineate the specific function and mechanism of the JNK-SAB-ROS activation loop. The contribution of the JNK-SAB-ROS activation loop in ferroptosis has been largely ignored. The results for the role of mitochondrial-derived ROS and structural/functional changes of mitochondria certainly support this.

Pyroptosis: Pyroptosis is a pro-inflammatory regulated cell death with necrotic morphology characterized at the late stage by the binding of cleaved gasdermin family proteins to the plasma membrane, followed by oligomerization to form membrane pores and release of inflammatory cytokines including interleukin (IL)-1β and IL-18 (Galluzzi et al., 2018). Long before, pyroptosis was referred to as caspase-1-mediated monocyte/macrophage death in immunity against intracellular pathogens and lipopolysaccharide (LPS) (Jorgensen and Miao, 2015). Pyroptosis has now been described in other mammalian cells including hepatocytes, endothelial cells, neurons, nephrons, and epithelial cells (Huang et al., 2020; Gaul et al., 2021; Shen et al., 2021; Luo et al., 2022). Depending on the tissue-specific expression of mammalian gasdermin genes (A-E and DFNB59), pyroptosis is involved in diseases including liver fibrosis, inflammatory bowel disease, asthma, multiple sclerosis, nephrotoxicity, lupus nephritis, cancer, and hearing loss (Gaul et al., 2021; Liu et al., 2021; Shen et al., 2021; Yu et al., 2021; Luo et al., 2022). Upon cleavage of gasdermins (A, B, C, or D), the N terminus of gasdermin (GSDM-NT) binds to acidic phospholipids on the inner side of the plasma membrane and oligomerizes to form pores in the plasma membrane, and downstream protein Ninjurin-1 (NINJ1) mediated the rupture of the membrane, facilitating pyroptosis (Evavold et al., 2018; Kayagaki et al., 2021; Liu et al., 2021; Xia et al., 2021). The critical step in gasdermin cleavage is caspase-1 activation in the canonical pathway and caspase-11 (4/5 in humans) activation in the non-canonical pathway (Willingham et al., 2007; Kayagaki et al., 2011). Recently, additional caspases (3 and 8) have been identified in specific conditions exposed to chemotherapeutic agents (Wang Y. et al., 2017; Rogers et al., 2017; Newton et al., 2019) and granzymes (A and B) in antitumor response (Zhang et al., 2020; Zhou et al., 2020). Thus, the involvement of caspases in apoptotic or pyroptotic cell death suggests the possible existence of a critical turning point to commit to one of the modes of cell death-apoptosis or pyroptosis.

Intriguingly, recent findings support the critical role of mitochondria in the regulation of pyroptosis (Huang et al., 2020; Evavold et al., 2021; Miao et al., 2023). Mitochondria-deficient ethidium bromide-treated macrophages are highly resistant to both canonical and non-canonical pyroptosis, suggesting the specific contribution of mitochondria to pyroptosis (Miao et al., 2023). In fact, the cleaved N-terminus of gasdermin D (GASDMD-NT) selectively binds to mitochondrial-specific membrane phospholipid, cardiolipin, which is mainly localized at the matrix side of IMM but is present in a small amount (3%–5%) in the OMM. GASDMD-NT affinity to cardiolipin is much higher than plasma membrane phospholipid, and mitochondria are targeted earlier than the plasma membrane. The direct mitochondrial targeting of GASDMD-NT, attenuation of mitochondrial respiration, increased ROS production, and membrane depolarization occur earlier than plasma membrane pore formation and are required to commit to the pyroptosis mode of cell death (Miao et al., 2023). Notably, mitochondrial antioxidant, Mito-TEMPO, prevents pyroptosis inducer-activated cell death but mitochondrial toxin, rotenone (at a low-lethal dose), which induces ROS alone is not sufficient to activate pyroptosis, suggesting an additional factor facilitating the exposure of cardiolipin to GASDMD-NT on mitochondria. Cardiolipin synthase 1 (CRLS1) in the matrix synthesizes cardiolipin which can be flipped from the matrix side of IMM to the outer side of IMM by phospholipid scramblase 3 (PLSCR3) and from there distributed to both sides of the OMM. Deletion of PLSCR3 completely prevents cardiolipin distribution to OMM and thus prevents pyroptosis inducer-activated cell death, demonstrating the absolute requirement of cardiolipin flipping to commit to pyroptosis. Of note, depolarization of IMM potential which triggers cardiolipin flipping is a plausible determinant to facilitate oligomerization of GASDMD-NT pores on mitochondria, leading to enhanced plasma membrane pore formation and cell rupture (Miao et al., 2023). The key question is how the mitochondrial depolarization occurs in pyroptosis. Mitochondrial membrane potential (MMP) is primarily maintained by mitochondrial respiration, which depends on NADH and ADP levels in the matrix. Recently, stress kinase (JNK) translocation and interaction with OMM protein SAB (SH3BP5) mediate the inhibition of mitochondrial respiration (Win et al., 2016), suggesting the possible involvement of SAB and P-JNK interaction in pyroptosis. In fact, ROS and the activated form of JNK (P-JNK) are upstream of caspase-3 activation in doxorubicin-induced GASDME cleavage and pyroptosis in breast cancer cells (Zhang et al., 2021). In addition, JNK is activated by inflammasome and high glucose in diabetic nephropathy (Qiao et al., 2018). Moreover, caspase 11 expression is upregulated by activated JNK-mediated transcription in macrophages of mouse models of enteropathogenic *Citrobacter rodentium* infection (Lupfer et al., 2014). Although the role of mitochondria translocated JNK in the regulation of pyroptosis was not yet assessed, JNK activation plays a role in enhanced inflammasome activation, increased caspase-11 expression and activation, caspase 3 activation, gasdermin cleavage, and pyroptosis.

Externalized cardiolipin signals several pathways that target mitochondria, such as mitophagy *via* autophagosome formation, apoptosis *via* Bid cleavage by activated caspase-8 promoting oligomerized BAX/BAK pore on OMM, and releasing cytochrome c. Therefore, the mitochondrial signals of the cell fate are overlapping, but the faster mitochondrial targeting of GASDMD-NT and subsequent targeting of GASDMD-NT to the plasma membrane commit the cells to pyroptosis mode of death, causing inflammatory responses. Recently, genome-wide screening identified the Ragulator–Rag complex through positive regulation of mTOR1 signaling as necessary for GSDMD pore formation. However, Rag and mTOR are not directly involved in GASDMD cleavage and pore formation, but mitochondrial ROS production is associated to facilitate GASDMD-NT pore formation on the plasma membrane (Evavold et al., 2021). This finding proposes that other activators of the mTOR pathway such as growth factors, glucose, cytokine receptors, and Toll-like receptor (TLR) ligands (Liu and Sabatini, 2020) could promote GASDMD oligomerization when the condition is associated with mitochondrial ROS production. In addition, GASDMD can be post-translationally modified by mitochondrial citric acid cycle intermediates (Humphries et al., 2020). Therefore, pyroptosis is not only a mode of cell death due to canonical and non-canonical inflammasomes but also could occur in metabolic stress in cells. Updates on pyroptosis mechanism and gasdermin-associated diseases have been thoroughly reviewed recently (Shi et al., 2017; Liu et al., 2021; Yu et al., 2021).

### SAB as a biomarker and therapeutic target

Chambers et al. reported investigations that led to the recognition of SAB as a biomarker and therapeutic target. As we discussed, the level of SAB expression in mitochondria determines the sustainability of P-JNK and cell death or disease severity. In the murine model, acetaminophen-induced liver injury is reduced in adult female mice because of lower basal expression of SAB in females than in males (Win et al., 2019). Progressive increased SAB expression is noted in the diet-induced progression of metabolic dysfunction-associated steatohepatitis (MASH, new nomenclature of NASH), and dialing up the level of P-JNK in the liver directly correlates with the level of SAB (Win et al., 2019; Win et al., 2021). In addition, the level of SAB expression predicts the chemotherapeutic drug sensitivity of various ovarian cancer cells (Chambers et al., 2015; Paudel et al., 2018). These intriguing results deserve further investigation of the clinical relevance of the SAB protein level and the mechanisms that regulate SAB expression.

SAB is expressed ubiquitously in various cells and tissues with high expression in the adrenal gland, fat, brain, heart, bone marrow, lymph node, and ovary. Chamber et al. first developed the KIM1 peptide (peptide sequence mimic to KIM1 amino acid sequence) designed to block SAB and P-JNK interaction in an *in vitro* binding assay system before the topology of SAB in mitochondria was characterized (Chambers et al., 2011a; Chambers and LoGrasso, 2011; Win et al., 2016). Membrane permeable form of KIM1 peptide in *in vitro* and *in vivo* models blocked JNK-mediated mitochondrial impairment, supporting the conclusion that the KIM1 peptide competitively prevents P-JNK binding to KIM2 because the KIM1 sequence has a higher affinity than KIM2 in binding assay (Chambers et al., 2011a; Chambers and LoGrasso, 2011). Furthermore, the KIM1 peptide prevents numerous models of JNK-mediated disease including 6-hydroxydopamine-induced toxicity in the brain, Parkinson’s disease, ischemia/reperfusion injury of the heart, and cardiotoxicity model. All these results suggest that the JNK-SAB-ROS activation loop is a feasible target for therapeutic development. In addition, targeting SAB expression dampens sustained JNK activation which offers therapeutic application (Win et al., 2021). KIM1 peptide can selectively block P-JNK binding to SAB without interfering with the JNK kinase activity (Chambers et al., 2011a; Win et al., 2014; Win et al., 2015) and has a specificity compared to the JIP-peptide which has a broader inhibitory action (Barr et al., 2002). However, the discovery of small molecules specifically targeting JNK and SAB interaction seems challenging. Alternatively, modulation of SAB gene expression may be possible using antisense oligonucleotides (Win et al., 2021). Importantly, there is no phenotype difference between wild type *versus* absence of hepatic SAB (knockout or knockdown of SAB in the liver). Modulating SAB expression may be a promising therapeutic approach in chronic diseases and cancer treatment.

## Conclusion

The JNK-SAB-ROS activation loop was uncovered recently, but the importance of the loop in the regulation of various modes of cell death has not been incorporated. This review has proposed the possible involvement of JNK and SAB interaction in ferroptosis and pyroptosis. In fact, the JNK-SAB-ROS activation loop can be modulated by crosstalk regulations, such as increased expression of DUSP *via* the p38a/MK2 pathway (Lalaoui et al., 2016), delayed antioxidant GSH recovery by activated JNK targeting, and degradation of GCLC (Win et al., 2023), and extensive protein nitration and lipid peroxidation by increased uptake of Fe^2+^ released from lysosomes (Hu and Lemasters, 2020; Adelusi et al., 2022). The liver is a unique organ with the capability to synthesize GSH, which is a major antioxidant system preventing cell death (Ookhtens and Kaplowitz, 1998; Yuan and Kaplowitz, 2009). Therefore, upregulation of NRF2 (*NFE2L2*) in oxidative stress and NF-κB (*NFKB1*) mediated the expression of mitochondrial antioxidants; superoxide dismutase 2 (SOD2) could interfere with the cell death pathways in hepatocytes compared to other organs and cells (Morgan and Liu, 2011; Han et al., 2013). However, the crucial role of JNK–SAB interaction was reported in cardiotoxicity, ischemia/reperfusion cardiac injury, neurotoxicity of the brain, Parkinson’s disease model, and neuronal activity of the brain (Chambers et al., 2011b; Chambers et al., 2013a; Chambers et al., 2013b; Chambers et al., 2017; Sodero et al., 2017). Therefore, interfering with the JNK-SAB-ROS pathway directly or indirectly modulates the mode of cell death.

## Figures and Tables

**Table:** 

**AP-1**	activating protein-1
**APAP**	acetaminophen
**ASK1**	apoptosis signal-regulating kinase 1
**AMPK**	AMP-activated kinase
**BCL2**	B-cell lymphoma 2
**CARD6**	caspase recruitment domain protein 6
**cFLIP**	cellular FLICE-like inhibitory protein
**CRLS1**	cardiolipin synthase 1
**ER**	endoplasmic reticulum
**GalN**	galactosamine
**GSH**	glutathione
**GSDM**	gasdermin
**GTPases**	guanosine triphosphate hydrolase
**IMM**	inner mitochondrial membrane
**ITCH**	itchy E3 ubiquitin protein ligase
**JIP**	JNK-interacting protein
**JNK**	c-Jun NH2-terminal kinase
**KIM**	kinase interaction motif
**LPS**	lipopolysaccharide
**MAPK**	mitogen-activated protein kinase
**MASH**	metabolic dysfunction-associated steatohepatitis
**MEF**	mouse embryonic fibroblast
**Mito-TEMPO**	mitochondria-targeted antioxidant
**MLK**	mixed lineage kinase
**MMP**	mitochondrial membrane potential
**MPTP**	mitochondrial permeability transition pore
**NADPH**	nicotinamide adenine dinucleotide phosphate hydrogen
**NCC**	N-terminal coiled-coil
**NCoR1**	nuclear receptor corepressor 1
**NLRP3**	NLR family pyrin domain containing 3
**OMM**	outer mitochondrial membrane
**PLSCR3**	phospholipid scramblase 3
**PPARα**	peroxisome proliferator-activated receptor alpha
**PUMA**	p53-upregulated modulator of apoptosis
**RAB-11**	Ras-related GTP-binding protein Rab11
**RAC**	Ras-related C3 botulinum toxin substrate
**REI-1**	guanine nucleotide exchange factor rei-1
**ROS**	reactive oxygen species
**SAB**	SH3BP5
**SOD2**	superoxide dismutase 2
**SRC**	proto-oncogene tyrosine-protein kinase Src
**TAK1**	transforming growth factor-β (TGF-β)-activated kinase 1
**TNF**	tumor necrosis factor
**TRAF**	tumor necrosis factor receptor-associated factor
